# Reply to Nicholas et al. Using a ResNet-18 Network to Detect Features of Alzheimer’s Disease on Functional Magnetic Resonance Imaging: A Failed Replication. Comment on “Odusami et al. Analysis of Features of Alzheimer’s Disease: Detection of Early Stage from Functional Brain Changes in Magnetic Resonance Images Using a Finetuned ResNet18 Network. *Diagnostics* 2021, *11*, 1071”

**DOI:** 10.3390/diagnostics12051097

**Published:** 2022-04-27

**Authors:** Modupe Odusami, Rytis Maskeliūnas, Robertas Damaševičius, Tomas Krilavičius

**Affiliations:** 1 Department of Multimedia Engineering, Kaunas University of Technology, 44249 Kaunas, Lithuania; modupe.odusami@ktu.edu (M.O.); rytis.maskeliunas@ktu.lt (R.M.); 2 Department of Applied Informatics, Vytautas Magnus University, 44248 Kaunas, Lithuania; tomas.krilavicius@vdu.lt

We have studied the manuscript of Nicholas et al. [1] very attentively; here are our comments:

The authors have used a different dataset (ADNI-3, rather than ADNI-2 used in [2]). The protocols of ADNI-2 and ADNI-3 datasets are not fully consistent [3]. The ADNI MR data set includes a wide range of scanner platforms; however, there has been a broad gap between older MRI systems and the state-of-the-art systems within each vendor’s product line. In ADNI-3, the “ADNI 3 Basic” and “ADNI 3 Advanced” protocols were used. The authors failed to mention if the images they used were made using a protocol compatible with ADNI-2. The dMRI spatial resolution was improved between ADNI-2 and ADNI-3 by reducing the voxel size from 2.7 × 2.7 × 2.7 mm to 2.0 × 2.0 × 2.0 mm [4]. This may have influenced the results. Moreover, the classification results among these studies are not directly comparable, because they differ in terms of the sets of participants.

We fully agree that the replication of important findings by multiple independent investigators is fundamental to the accumulation of scientific evidence [5]. Deep learning network models are notoriously known for being difficult to replicate, even if the same sets of parameters are used. The training of neural network models is not deterministic, so the models are likely to produce differing results [6]. The strive of the authors to precisely replicate the results may not be achievable.

1. Considering the description of the training process described in their manuscript, we have tried our model on the ADNI-3 dataset using both cross-validation procedures used by To et al., However, we failed to replicate their results (see the result Table 1 and Figure 1).
2. Our result is not exceptional. In fact, it is in line with the state-of-the-art studies, which achieved a similar high performance in the ADNI dataset by using 2D CNN, ResNet-18 [7] and custom CNN [8], as well as in other datasets such as OASIS [9,10]. We are somewhat puzzled as to why the performance reported by To et al. on the ADNI dataset is so low.

## Figures and Tables

**Figure 1:**
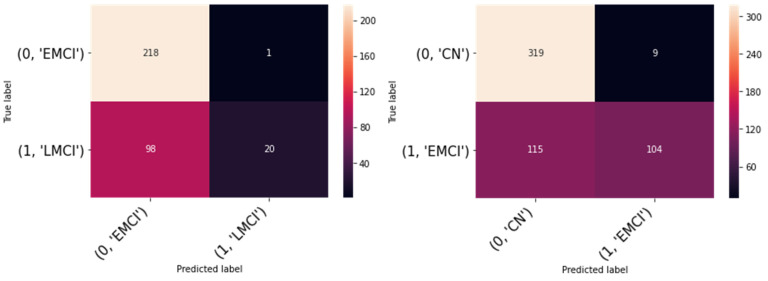
Confusion matrices of replicated result.

**Table 1:** Replicated results.

Binary Classes	Accuracy (%)	Sensitivity (%)	Specificity (%)
EMCI vs. LMCI	70.62	68.98	95.23
CN vs. EMCI	77.30	73.50	92.03
